# The interaction between changes of muscle activation and cortical network dynamics during isometric elbow contraction: a sEMG and fNIRS study

**DOI:** 10.3389/fbioe.2023.1176054

**Published:** 2023-04-25

**Authors:** Xiaohan Wang, Zichong Luo, Mingxia Zhang, Weihua Zhao, Songyun Xie, Seng Fat Wong, Huijing Hu, Le Li

**Affiliations:** ^1^ Institute of Medical Research, Northwestern Polytechnical University, Xi’an, China; ^2^ Faculty of Science and Technology, University of Macau, Taipa, China; ^3^ Hospital of Northwestern Polytechnical University, Xi’an, China; ^4^ School of Electronics and Information, Northwestern Polytechnical University, Xi’an, China

**Keywords:** fNIRS, electromyography, brain network, fuzzy approximate entropy, muscle isometric contraction

## Abstract

**Objective:** The relationship between muscle activation during motor tasks and cerebral cortical activity remains poorly understood. The aim of this study was to investigate the correlation between brain network connectivity and the non-linear characteristics of muscle activation changes during different levels of isometric contractions.

**Methods:** Twenty-one healthy subjects were recruited and were asked to perform isometric elbow contractions in both dominant and non-dominant sides. Blood oxygen concentrations in brain from functional Near-infrared Spectroscopy (fNIRS) and surface electromyography (sEMG) signals in the biceps brachii (BIC) and triceps brachii (TRI) muscles were recorded simultaneously and compared during 80% and 20% of maximum voluntary contraction (MVC). Functional connectivity, effective connectivity, and graph theory indicators were used to measure information interaction in brain activity during motor tasks. The non-linear characteristics of sEMG signals, fuzzy approximate entropy (fApEn), were used to evaluate the signal complexity changes in motor tasks. Pearson correlation analysis was used to examine the correlation between brain network characteristic values and sEMG parameters under different task conditions.

**Results:** The effective connectivity between brain regions in motor tasks in dominant side was significantly higher than that in non-dominant side under different contractions (*p* < 0.05). The results of graph theory analysis showed that the clustering coefficient and node-local efficiency of the contralateral motor cortex were significantly varied under different contractions (*p* < 0.01). fApEn and co-contraction index (CCI) of sEMG under 80% MVC condition were significantly higher than that under 20% MVC condition (*p* < 0.05). There was a significant positive correlation between the fApEn and the blood oxygen value in the contralateral brain regions in both dominant or non-dominant sides (*p* < 0.001). The node-local efficiency of the contralateral motor cortex in the dominant side was positively correlated with the fApEn of the EMG signals (*p* < 0.05).

**Conclusion:** In this study, the mapping relationship between brain network related indicators and non-linear characteristic of sEMG in different motor tasks was verified. These findings provide evidence for further exploration of the interaction between the brain activity and the execution of motor tasks, and the parameters might be useful in evaluation of rehabilitation intervention.

## 1 Introduction

Human execute motor tasks and promote individuals to complete activities of daily life and social interaction (Shumway-Cook and Woollacott, 2014). The completion of motor tasks consists of corresponding muscle contractions and stretches, so it is important to generate accurate motor control, which is considered to be the fundamental factor of movement (Yokoyama et al., 2019). Previous studies, such as electroencephalography (EEG) and functional magnetic resonance imaging (fMRI), have found that upper limb movement is regulated by corresponding regions of the cerebral cortex (Long et al., 2016; Lin et al., 2017). It is believed that the relationship between the muscle contractions and correlated cerebral hemodynamic is essential to the understanding of motor control such as the planning and execution of motor tasks (Winter 2009; Zhou et al., 2022). However, the low spatial resolution of EEG and the high costs of fMRI limit the development of relevant motor studies and it is also very challenging to combine the fMRI with human daily activity due to the strict requirement of subject’s stabilization during the scan.

Functional Near-infrared Spectroscopy (fNIRS) is a non-invasive brain functional imaging technology based on optical principles (Ferrari and Quaresima, 2012) and it has the advantages of being safe and non-invasive, easy to move, anti-motion and electromagnetic interference, high spatial resolution, and allowing long-term monitoring (Herold et al., 2018). Neuroscience research has highlighted the importance of brain networks between different brain regions in motor tasks (Felleman and Van Essen, 1991). fNIRS technique facilitated the investigation of elucidating cerebral hemodynamic changes during muscle contraction motor tasks (Zimmermann et al., 2013). Functional connectivity, effective connectivity and graph theory analysis between different brain regions have been applied to the study of neural function and motor function. Xu et al. found that brain network indicators, including clustering coefficient and global efficiency, could be used to evaluate the rehabilitation effects of post-stroke exercise training (Xu et al., 2022). However, it remains unclear whether the connections between the cerebral cortex are related to motor performance during execution of motor tasks, so the relationship between muscle excitability and brain network changes warrants further investigation.

Recording of muscle activity by electromyography (EMG) from skin surface, so called sEMG, has proved to be useful for evaluating central and peripheral determinants of motor function (Donaldson et al., 2003). The analysis of sEMG signals can explore the activity state of muscle tissue and the control mechanism of the nerve center under different task states, which is of great significance for the training and evaluation of rehabilitation intervention (Vigotsky et al., 2017). sEMG is characterized by high complexity and chaos (Hong-Chun et al., 2005), so it is imperative to use non-linear characteristic analysis methods to analyze sEMG signals (Sun et al., 2014). Entropy indicates the complicated properties of the researched signal with a simple yet effective approach and high consistency in order to analyze biological signals such as sEMG. Chen et al. proposed fuzzy approximate entropy (fApEn) based on the entropy theory by introducing the idea of a fuzzy set (Chen et al., 2007). Previous studies have shown that fApEn can be used as an indicator of changes in the complexity of sEMG signals to determine the alterations in motor control during task execution (Ao et al., 2015; Deng et al., 2021). However, the relationship between changes in muscle complexity and brain network during task execution remains unclear. Understanding the coupling mechanism between the cerebral cortex and muscle is essential in the study of human motor control (Bao et al., 2021; Colamarino et al., 2021).

Therefore, in current study, fNIRS and sEMG were used to investigate the interaction between brain region activation and peripheral muscle contraction in different motor control tasks. The connectivity parameters between brain regions in motor tasks (functional connectivity, effective connectivity and graph theory indicators) and the non-linear analysis of muscle activation from fApEn were conducted. We hypothesize that the brain network connectivity is correlated to the efficiency of peripheral motor control, and the findings of current study may help to understand the underlying mechanism of corticomuscular coupling.

## 2 Methods

### 2.1 Participants

Twenty-one healthy subjects (mean age: 23.86 ± 1.35, 12 males and 9 females) were recruited from Northwestern Polytechnical University for this study. All of the subjects were right-hand dominant with normal vision and did not have any history of epilepsy or other psychiatric disorders according to the Edinburgh Handedness Inventory. This study was approved by the local institutional ethics committee and was registered in the Chinese Clinical Trial Registry (ChiCTR2200057839). All participants understood the content of the experiment and signed the informed consent before participating in the study. In addition, subjects were allowed enough time to rest to ensure their attention during the experiment.

### 2.2 Experimental procedures

**FIGURE 1:**
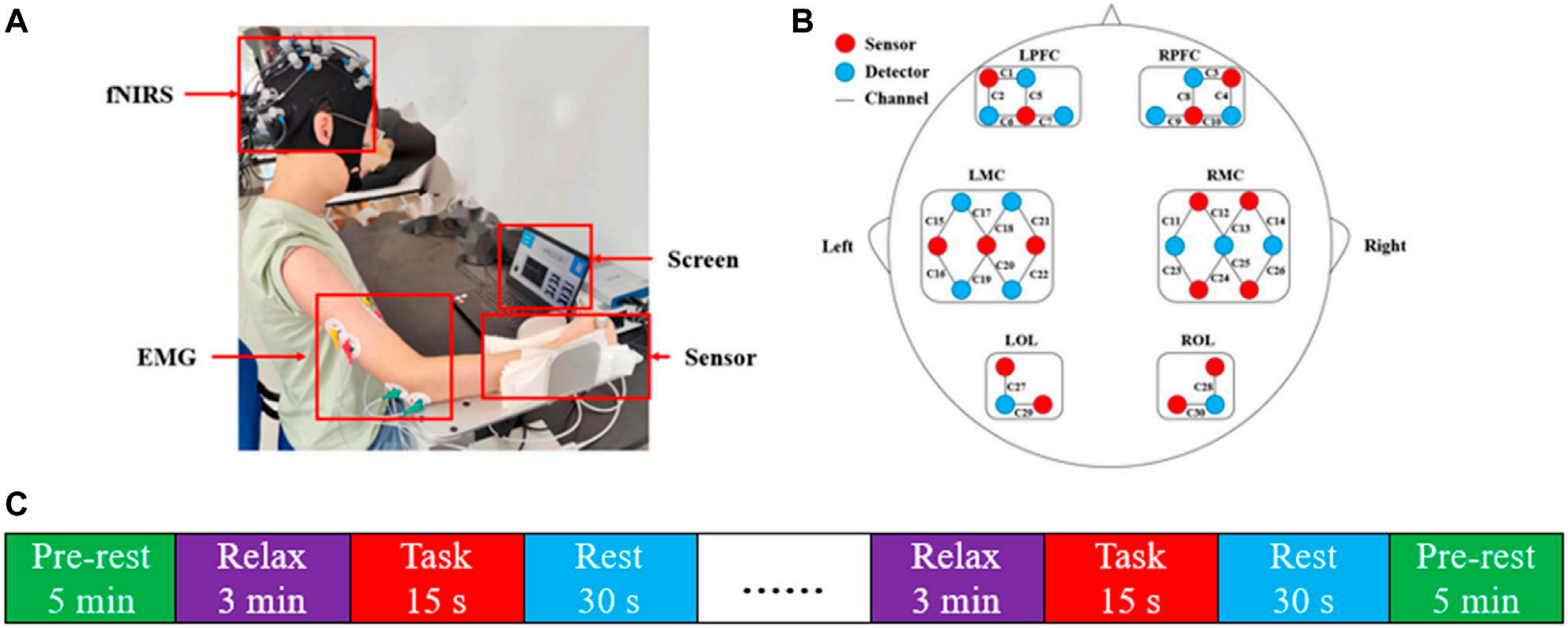
The experimental design of the evaluation test. **(A)** Experimental set-up. **(B)** The layout of fNIRS channels. The red dots represented sources, and the blue dots represented detectors. The 15 sources and 15 detectors constitute 30 channels, overlaying six brain regions: LPFC, RPFC, LMC, RMC, LOL, and ROL. **(C)** Experiment procedure. Each trial consisted of a combination of a 15 s task and a 30 s rest and was repeated five times in each condition.

Before the data collection, the operator explained the entire procedure to each participant until they fully understood. Figure 1A shows the experimental setup in this study. In the process of experiment, participants were asked to sit on a chair, facing the computer screen. The subject’s forearm was fixed to the slot of the homemade handrail. Their elbows were flexed at 90°, and their shoulders were turned at 90° of abduction. All participants practiced muscle contraction to achieve the target values in response to visual feedback. The visualization experiment program was provided by a custom LabVIEW (LabVIEW 2012; National Instruments, Austin, TX, United States) program. First, the subjects were asked to produce consecutive maximum voluntary contractions (MVC) lasting 5 s each, repeating three times with a 2-min rest interval to avoid muscle fatigue. Secondly, subjects completed isometric elbow contraction under visual feedback regulation. Each trial included two conditions, 20% MVC and 80% MVC, repeated five times each. Subjects were required to complete 20 trials in both the dominant and non-dominant sides. Each trial consisted of a 3-min relaxation phase, a 15-s task phase, and a 30-s rest phase. The experimental procedure is shown in Figure 1C.

### 2.3 Data acquisition

fNIRS signals with NIR wavelengths of 730 and 850 nm were collected using a multichannel fNIRS system (NirSmart, Danyang Huichuang Medical Equipment Co. Ltd., China) at a sampling rate of 11 Hz. Figure 1B demonstrates the usage of a standard cap with an international 10–20 system to reference the NIR probe layout. The distance between the source and the detector was 30 mm to ensure that the signal was detected from the brain’s gray matter. A total of 30 channels were used to cover the left and right prefrontal cortex (LPFC, RPFC), left and right motor cortex (LMC, RMC), and left and right occipital lobe (LOL, ROL). To collect the sEMG signals, the skin above the subjects’ muscle was scraped and cleaned with alcohol. Ag-AgCl bipolar electrodes were placed on the belly of muscle with a constant distance of 20 mm between electrodes. sEMG signals were recorded by a custom sEMG amplifier and sampled by a data converter at 1,000 Hz. fNIRS and sEMG were synchronized through the LabVIEW program. A two-channel sEMG amplifier with a gain of 5,000 was applied to record the sEMG signals. The acquisition rate of the sEMG signals was at 1,000 Hz, sampled by a 16-bit data converter (DAQ-6341, National Instruments, Austin, TX, United States). A LabVIEW program was used to monitor and save the raw sEMG signals. To synchronize the EMG and fNIRS devices, timestamp signals were sent simultaneously to two devices at the beginning and end of each task recording using the same computer that presented the task and visual feedback, and each “go” cue was stored by each device as its time reference (Ortega et al., 2020; Wang et al., 2020). In addition, EMG and FNIRS signals were aligned using the synchronization program developed in LabView (Jian et al., 2021).

### 2.4 Data analysis

#### 2.4.1 Data preprocess

In this experiment, raw sEMG signals of BIC and TRI were first filtered with a fourth-order bandpass Butterworth filter (20–450 Hz) and then filtered with a notch filter (50 Hz). For the preprocessing of fNIRS signal, the original fNIRS signal was first converted into the hemodynamic signal by the modified bill-lambert law. Each fNIRS channel could obtain an oxygenated hemoglobin (HbO_2_) concentration signal and a deoxyhemoglobin (HbR) concentration signal. Since the HbO_2_ signal showed a better signal noise ratio than the HbR signal, the HbO_2_ signal was chosen as a marker of cortical activation, and the task-related HbO_2_ concentration was calculated (Ye et al., 2009). After removing invalid channels and motion artifacts, the HbO_2_ signal was bandpass Butterworth filtered (0.01–0.2 Hz) to remove physiological signal interference, including interference caused by heartbeat and respiratory activity.

#### 2.4.2 EMG data analysis

Off-line EMG signals were digitally filtered by a zero-phase-shift fourth-order Butterworth bandpass filter with 10–490 Hz and a notch filter with a notch point at 50 Hz to cancel noise and power-line interference. Manual visual inspection was performed on each trial to determine segments of the 5-s EMG signal, with a consistent, steady flat response after RMS-smoothing.

##### 2.4.2.1 fApEn of EMG

Entropy is an effective non-linear method to represent physiological signals (Ao et al., 2015). The increase of entropy indicates the increase of complexity and the decrease of regularity of motor tasks. fApEn analysis was performed to calculate the complexity of EMG signal for each chopped segment. The theory of fApEn has been developed in previous studies so that a brief of the algorithm is described here (Xie et al., 2010; Sun et al., 2014):

For the time series T containing N EMG signal data point {e(i): 1 ≤ i ≤ N}, the following vector sequence can be formed as:

$$S_{i}^{m}=e\left(i\right),e\left(i+1\right),\cdots e\left(i+m-1\right)-e0\left(i\right)$$ (1)

where $S_{i}^{m}$ represents m consecutive e values, commencing with the *i*th point. $e0\left(i\right)$ is the baseline of the vector to be removed:

$$e0\left(i\right)=\frac{1}{m}\sum_{j=0}^{m-1}e\left(i+j\right)$$ (2)

Then, the distance $d_{ij}^{m}$ between two vectors $S_{i}^{m}$ and $S_{j}^{m}$ is defined as:

$$d_{ij}^{m}=d\left[S_{i}^{m},S_{j}^{m}\right]=\begin{array}{c} \max\\ k\in \left(0,m-1\right) \end{array}\left|\left(e\left(i+k\right)-e0\left(i\right)-\left(e\left(j+k\right)-e0\left(j\right)\right)\right.\right|$$ (3)

The similarity degree of $d_{ij}^{m}$ between $S_{i}^{m}$ and $S_{j}^{m}$ can be determined by an ambiguity function $\mu \left(d_{ij}^{m},n,r\right)$ , where n and r represent the width and gradient parameters of the boundary of the exp function respectively.

Dijmn,r=μdijm,n,r=exp⁡(−dijmnr) (4)

The function $\phi^{m}$ is defined as:

$$\phi^{m}\left(n\right)=\frac{1}{N-m+1}\sum_{i=1}^{N-m+1}\ln\left(\frac{1}{N-m-1}\sum_{j=1,j\neq i}^{N-m+1}D_{ij}^{m}\right)$$ (5)

Repeatedly, the function $\phi^{m+1}$ can be defined as:

$$\phi^{m+1}\left(n\right)=\frac{1}{N-m+1}\sum_{i=1}^{N-m+1}\ln\left(\frac{1}{N-m-1}\sum_{j=1,j\neq i}^{N-m+1}D_{ij}^{m+1}\right)$$ (6)

Finally, the function of fApEn of the EMG signal can be expressed as:

$$fApEn\left(m,r,n,N\right)=\phi^{m}\left(r\right)-\phi^{m+1}\left(r\right)$$ (7)

##### 2.4.2.2 Co-contraction index estimation

Co-contraction Index (CCI) is the EMG-based model to estimate the simultaneous contraction of antagonists and agonists during dynamic movement base. Several CCI calculation methods have been discussed in a previous study (Souissi et al., 2017). The method proposed by winter was applied in this study (Winter, 2009).

$$\%CCI=\frac{2\times common\;Area}{Area\;A+Area\;B}\times 100$$ (8)

Area A and Area B represent the area under the enveloped EMG antagonists and agonists curve, and the common area is the overlap area between Area A and Area B. Trapezoidal method is suggested for the integral calculation to estimate the areas. The CCI should vary from 0 to 100, where 0 indicates no overlap of the two EMG envelopes and 100 indicates that both muscles were fully activated to 100% MVC during the trial.

#### 2.4.3 fNIRS data analysis

The general linear model is a standard linear estimation method commonly used to extract hemodynamic responses from fNIRS data to examine brain-activated regions (Zhou et al., 2022). NirSpark software was used to process the original data to obtain the data on brain activation and functional connectivity strength under different task conditions (https://openfnirs.org/). After brain activation analysis, brain network analysis was performed to explore connectivity between brain regions further (Friston et al., 2013). Thus, there are dynamic interactions between each functional brain region to coordinate and respond to specific environmental and task demands.

Finally, common brain network indicators, including node-local efficiency and clustering coefficient, were calculated to characterize the functional connectivity of brain regions (http://www.brain-connectivity-toolbox.net) (Bullmore and Sporns, 2009; Rubinov and Sporns, 2010). The clustering coefficient measures network isolation and global efficiency index network integration (Wang et al., 2019). In contrast, node-local efficiency represents the ability to integrate neighboring nodes of a given node corresponding to brain regions (Yuan et al., 2022).

#### 2.4.4 Statistical analysis

All experimental data were statistically analyzed using IBM SPSS Statistics 26 (IBM Inc., WA, United States) and MATLAB R2021a (The MathWorks, United States) software. Data of functional connectivity were analyzed by independent sample *t*-test. The functional connectivity and effective connectivity of brain regions between different groups were analyzed by one-way ANOVA. Paired sample *t*-test was performed on the indexes such as clustering coefficient and node-local efficiency calculated in graph theory analysis as well as the EMG correlation characteristic values. Finally, Pearson correlation analysis was used to test the correlation between the activation intensity of each brain region and the fApEn of the EMG signal under different task conditions. *p* < 0.05 was considered statistically significant for all the statistical analyses and false discovery rate (FDR) correction was performed.

## 3 Results

### 3.1 Functional connectivity

**FIGURE 2:**
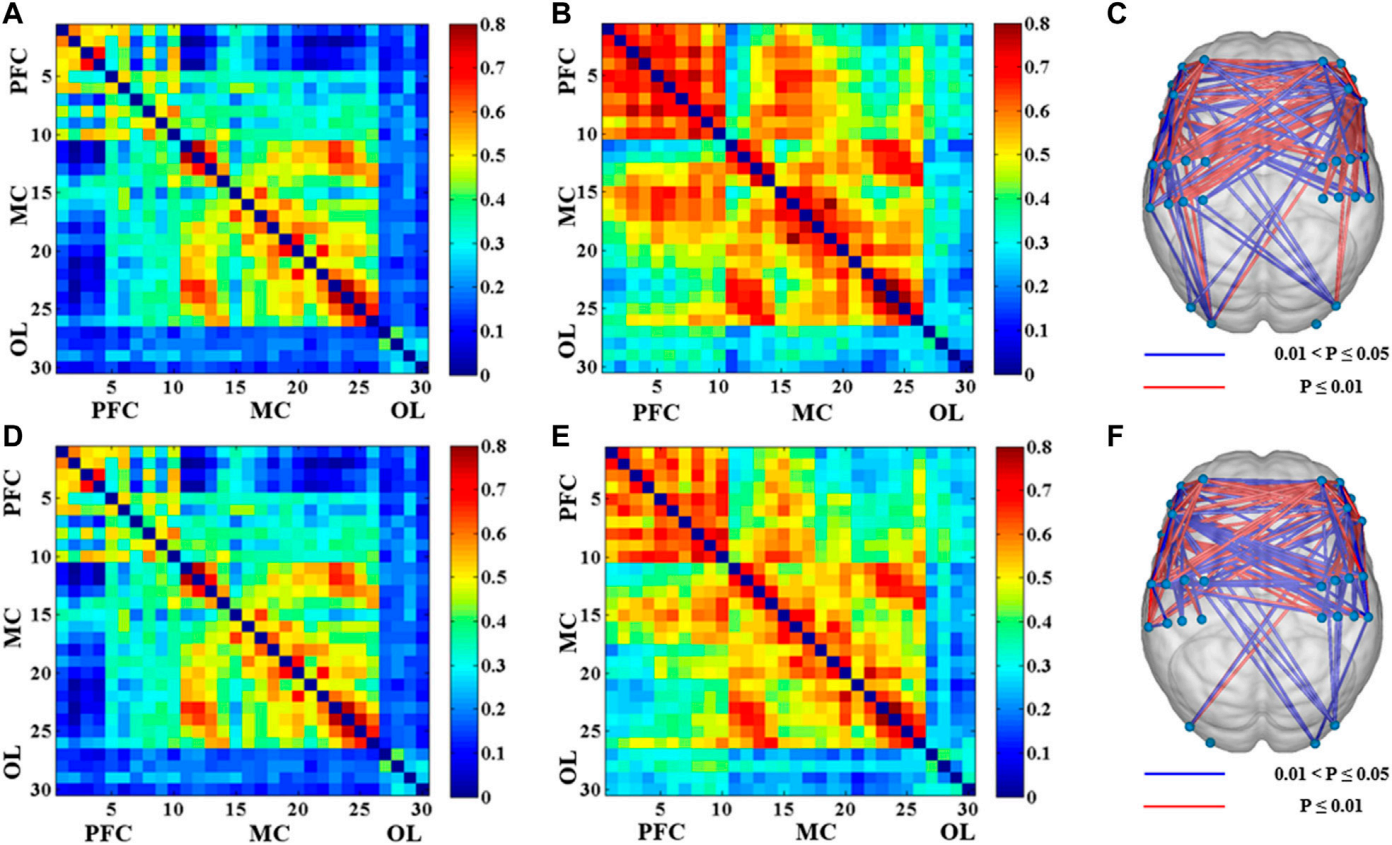
The results of the functional connectivity between 30 channels in different conditions (*p* < 0.05). **(A)** Correlation matrix between 30 channels under 20% MVC of the dominant side. **(B)** Correlation matrix between 30 channels under 80% MVC of the dominant side. **(C)** The significant difference of functional connectivity of the 80% MVC relative to the 20% MVC between 30 channels under the dominant side. **(D)** Correlation matrix between 30 channels under 20% MVC of the non-dominant side. **(E)** Correlation matrix between 30 channels under 80% MVC of the non-dominant side. **(F)** The significant difference of functional connectivity of the 80% MVC relative to the 20% MVC between 30 channels under the non-dominant side.

Figure 2 showed the functional connectivity matrix of brain channels of the dominant and non-dominant side during different levels of muscle contraction. Compared with the resting state, the functional connectivity between the corresponding channels in the contralateral and ipsilateral motor cortex was more robust under the 20% MVC condition of the dominant side (LMC-RMC: 0.44 ± 0.17). In contrast, the functional connectivity between other brain regions was weaker. Similarly, functional connectivity was stronger between the contralateral and ipsilateral motor cortex and between the contralateral and ipsilateral prefrontal cortex in the dominant side, with 80% MVC condition compared to the resting state (LPFC-RPFC: 0.63 ± 0.16; LMC-RMC: 0.52 ± 0.23) (Figures 2A–C). Under the 20% MVC condition of the non-dominant side, the functional connectivity between the corresponding channels of the contralateral motor cortex and the ipsilateral motor cortex was also stronger compared with the resting state (LMC-RMC: 0.44 ± 0.19). In comparison, the functional connectivity between other brain regions was weaker. Similarly, functional connectivity between the contralateral motor cortex and ipsilateral prefrontal cortex was also more significant in the 80% MVC of the non-dominant side compared to the resting state, as was functional connectivity between the contralateral prefrontal cortex and ipsilateral prefrontal cortex (LPFC-RPFC: 0.59 ± 0.17; LMC-RMC: 0.51 ± 0.20) (Figures 2D–F).

**FIGURE 3:**
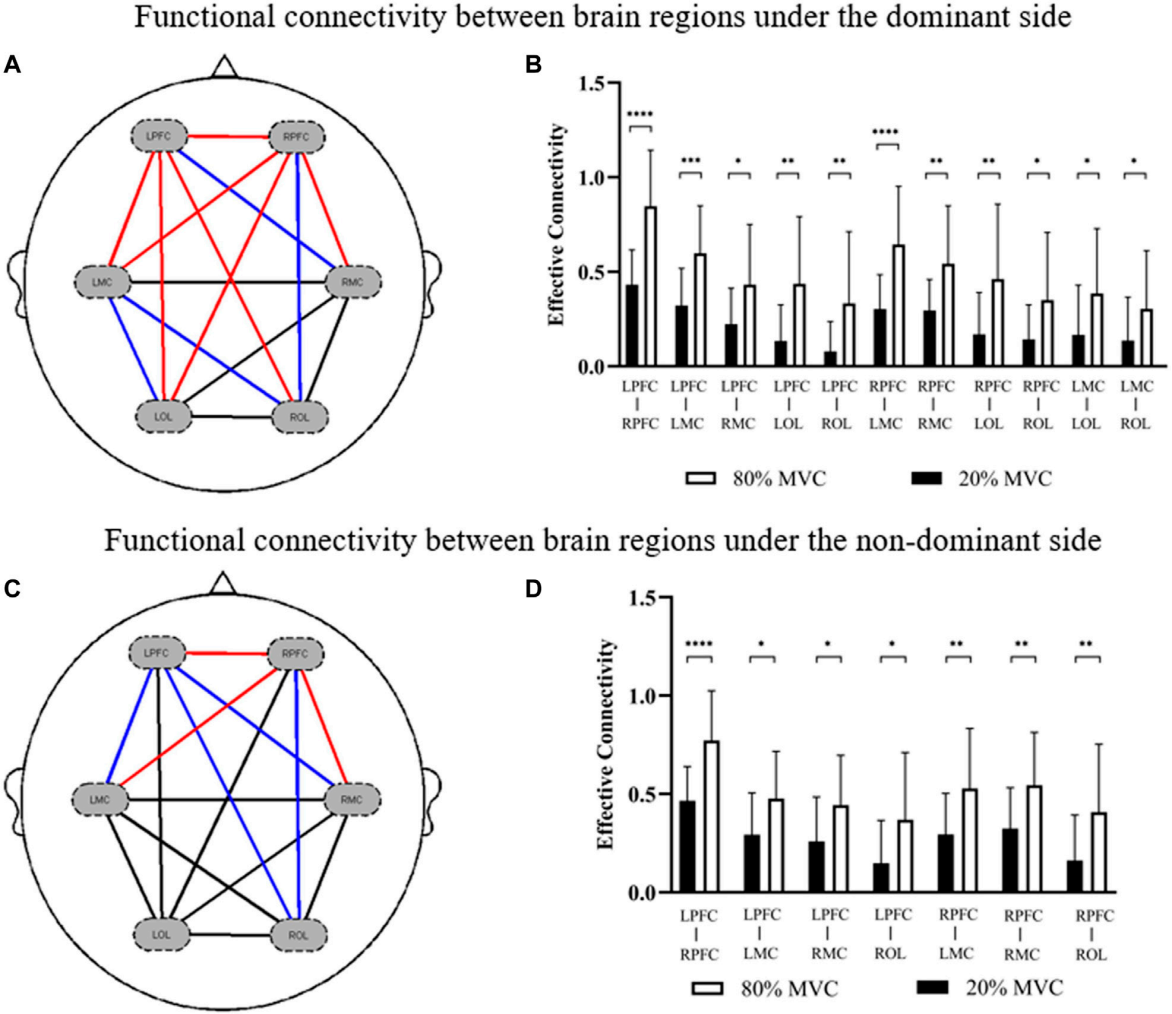
The results of significant differences in functional connections between brain regions in different conditions. **(A,B)** The change is represented by the functional connectivity of the 80% MVC relative to the 20% MVC under the dominant side. **(C,D)** The change is represented by the functional connectivity of the 80% MVC relative to the 20% MVC under the non-dominant side. The red line (*p* < 0.01) and blue line (0.01 < *p* < 0.05) indicate significant increase statistically.

The comparison results of functional connectivity of brain regions under different muscle contraction levels showed that the functional connectivity between the motor cortex and prefrontal cortex (LPFC-RMC: *p* = 0.017; RPFC-ROL: *p* = 0.020; LMC-LOL: *p* = 0.025; LMC-ROL: *p* = 0.049) was significantly enhanced under 80% MVC condition compared with 20% MVC condition of the dominant side, especially the functional connectivity between the contralateral motor cortex, contralateral prefrontal cortex and ipsilateral prefrontal cortex (LPFC-RPFC: *p* = 0.000; LPFC-LMC: *p* = 0.000; LPFC-LOL: *p* = 0.001; LPFC-ROL: *p* = 0.005; RPFC-LMC: *p* = 0.000; RPFC-RMC: *p* = 0.003; RPFC-LOL: *p* = 0.005) was significantly enhanced (Figures 3A, B). Similarly, functional connectivity between the motor cortex and prefrontal cortex (LPFC-LMC: *p* = 0.012; LPFC-RMC: *p* = 0.014; LPFC-ROL: *p* = 0.019; RPFC-ROL: *p* = 0.013) was significantly enhanced in the non-dominant side 80% MVC condition compared to 20% MVC, especially between contralateral motor cortex, contralateral prefrontal cortex, and ipsilateral prefrontal cortex (LPFC-RPFC: *p* = 0.000; RPFC-LMC: *p* = 0.009; RPFC-RMC: *p* = 0.006) (Figures 3C, D).

### 3.2 Effective connectivity

**FIGURE 4:**
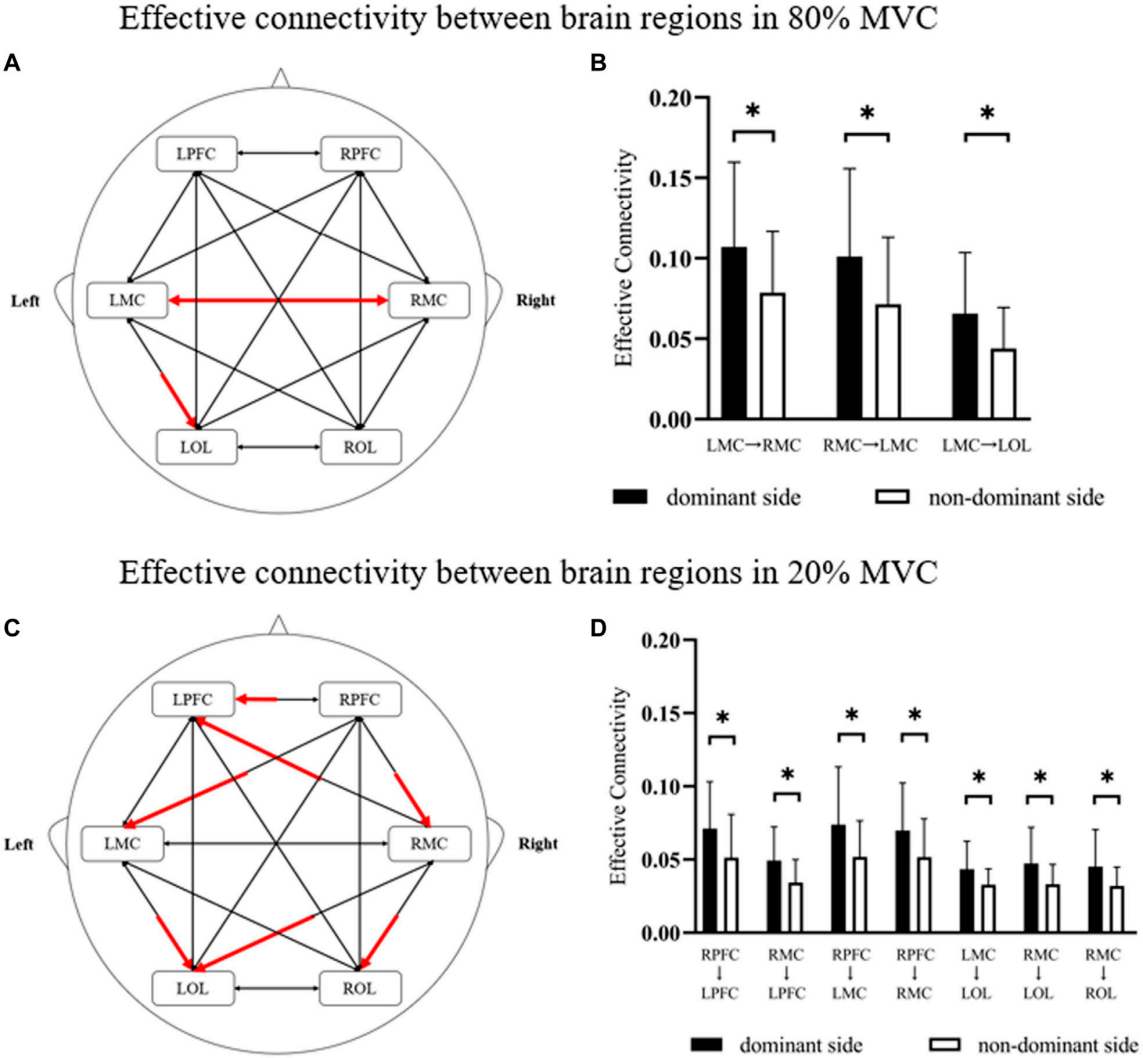
The results of significant differences in effective connectivity between brain regions in different conditions. **(A,B)** The change is represented by the effective connectivity of the dominant side relative to the non-dominant side under 80% MVC. **(C,D)** The change is represented by the effective connectivity of the dominant side relative to the non-dominant side under 20% MVC. The red line indicates a significant increase statistically (*p* < 0.05), where * indicates *p* < 0.05.

Figure 4 showed the effective connectivity between the different brain regions of the dominant and non-dominant sides under the same force conditions. Under the 80% MVC condition, the effective connectivity between other brain regions of the dominant and non-dominant sides showed that the effective connectivity was significantly enhanced under the dominant side condition from RMC to LMC (*p* = 0.046), from LMC to the RMC (*p* = 0.049), and from LMC to LOL (*p* = 0.032) (Figures 4A, B). Under the 20% MVC condition, the effective connectivity between different brain regions of the dominant and non-dominant sides showed that the effective connectivity from RPFC to LRPC (*p* = 0.039), from RPFC to LMC (*p* = 0.033), from RPFC to RMC (*p* = 0.047), from RMC to LPFC (*p* = 0.016), from RMC to LOL (*p* = 0.022), from RMC to LOL (*p* = 0.035) and from LMC to LOL (*p* = 0.028) was significantly enhanced under the dominant side condition (Figures 4C, D).

### 3.3 Brain network analysis

**FIGURE 5:**
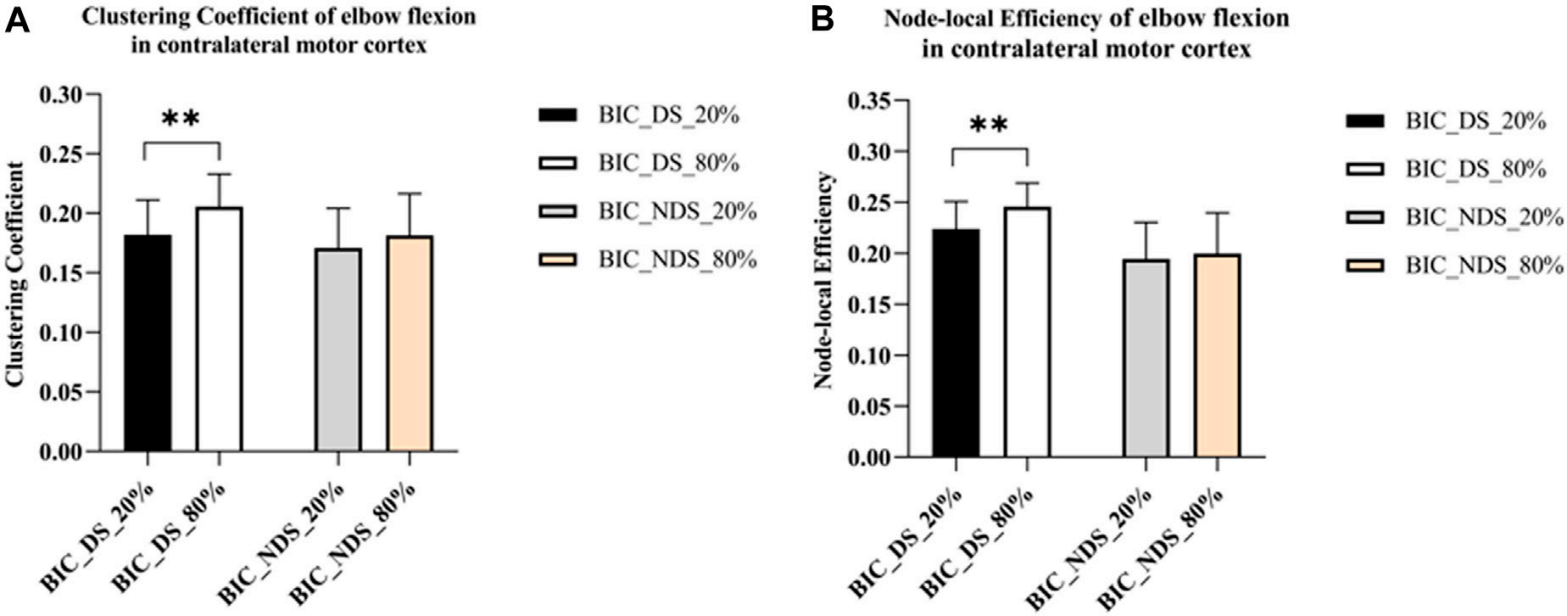
Significant global and local metric changes of elbow flexion in the contralateral motor cortex. **(A)** Global metrics results of the clustering coefficient (ΔHbO_2_). **(B)** Local metrics results of the node-local efficiency (ΔHbO_2_), where ** indicates *p* < 0.01. Abbreviation: DS, dominant side; NDS, non-dominant side.

As shown in Figure 5, only significant differences were observed in the clustering coefficient (*p* = 0.008) and node-local efficiency (*p* = 0.006) of the contralateral motor cortex under different force conditions of the dominant side. However, no significant difference was observed in the contralateral motor cortex under other force conditions of the non-dominant side. But the clustering coefficient of the dominant side under the same conditions was higher than those of the non-dominant side (C _dominant side_80%_ = 0.21; C _non-dominant side_80%_ = 0.18; C _dominant side_20%_ = 0.18; C _non-dominant side_20%_ = 0.17). Similarly, the node-local efficiency of the dominant side under the same conditions was higher than that of the non-dominant side (E _dominant side_80%_ = 0.25; E _non-dominant side_80%_ = 0.20; E _dominant side_20%_ = 0.22; E _non-dominant side_20%_ = 0.19). The results showed that the information connection efficiency was higher in the brain network of the contralateral motor cortex under 80% MVC condition of the dominant side. The information connection efficiency was lower in the brain network of the contralateral motor cortex with the non-dominant side.

### 3.4 EMG analysis

**FIGURE 6:**
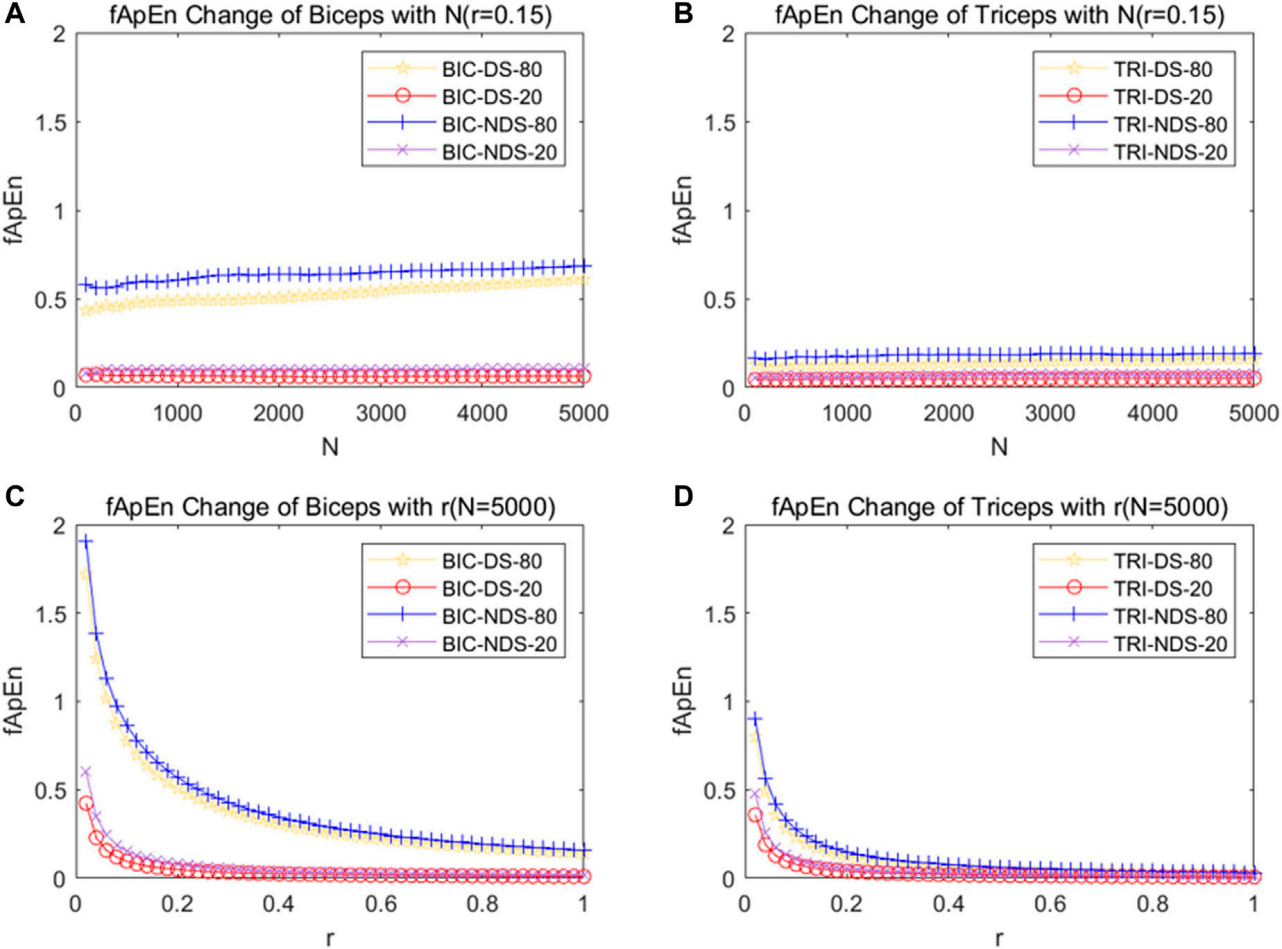
**(A)** Change of fApEn of BIC with the change of window N. **(B)** Change of fApEn of TRI with the change of window N. **(C)** Change of fApEn of BIC with the change of tolerance r. **(D)** Change of fApEn of TRI with the change of tolerance r. EMG signals were picked out from four different conditions from a subject. Abbreviation: DS, dominant side; NDS, non-dominant side.

**FIGURE 7:**
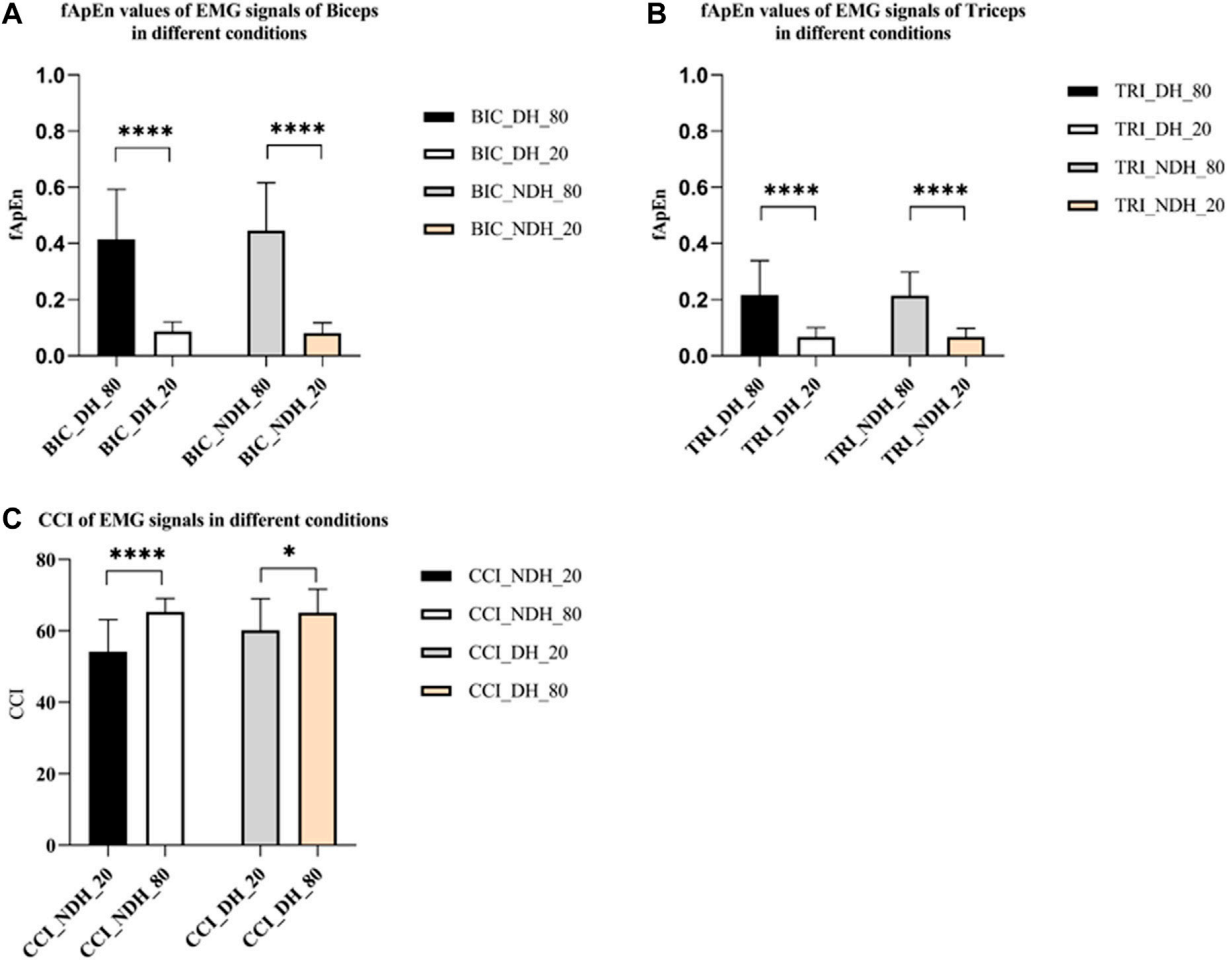
**(A)** fApEn values of EMG signals of BIC. **(B)** fApEn values of EMG signals of TRI. **(C)** The CCI of EMG signals in different conditions. The significant difference between different conditions was represented by *p < 0.05 and ****p < 0.0001. Abbreviation: DS, dominant side; NDS, non-dominant side.

Figures 6A, B depicted the fApEn of the EMG signal extracted from the BIC and TRI under different conditions as the window N increases from 100 points to 5,000 points in the step of 100 points size. Figures 6C, D respectively depicted the fApEn of the EMG signal when the admissible window r is increased from 0.02 to 1 at a step r of 0.02 for the picked EMG signal of BIC and TRI under four different conditions, respectively. Combined with the results reported in previous studies and the test results in this study, N was set as 5,000, r was fixed as 0.15, and the five repeated tests were averaged. Comparing the fApEn of EMG signals of the BIC, the results showed that fApEn under the condition of 80% MVC was significantly higher than that under the condition of 20% MVC, no matter it was dominant or non-dominant sides (*p* < 0.0001) (Figure 7A). Comparing the fApEn of EMG signals of the TRI, the results showed that the value under the 80% MVC condition was significantly higher than that under the 20% MVC condition, whether it was the dominant or non-dominant sides (*p* < 0.0001) (Figure 7B). The results showed that in the non-dominant side condition, the CCI of 80% MVC was significantly higher than that of 20% MVC (*p* = 0.000) (Figure 7C). Similarly, in the dominant side condition, the CCI of 80% MVC was significantly higher than that of 20% MVC (*p* = 0.048) (Figure 7C).

### 3.5 Correlation analysis

**FIGURE 8:**
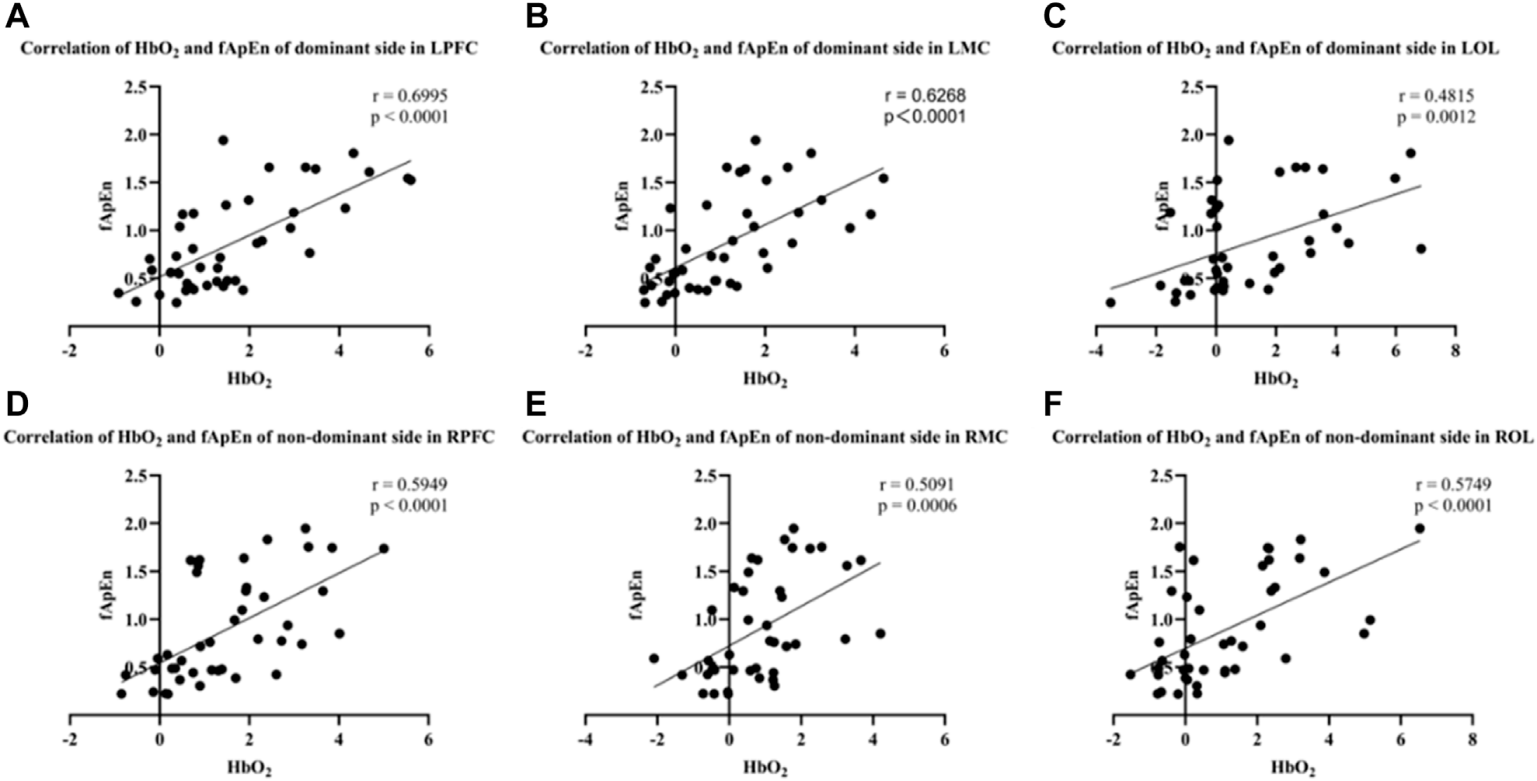
**(A–C)** Correlation of HbO_2_ and fApEn of the dominant side in LPFC (r = 0.6995, *p* < 0.0001), LMC (r = 0.6268, *p* < 0.0001) and LOL (r = 0.4815, *p* = 0.0012). **(D–F)** Correlation of HbO_2_ and fApEn of the non-dominant side in RPFC (r = 0.5945, *p* < 0.0001), RMC (r = 0.5091, *p* = 0.0006) and ROL (r = 0.5749, *p* < 0.0001).

As shown in Figure 8, fApEn of EMG signals in both dominant and non-dominant conditions was significantly correlated with activation in the contralateral prefrontal cortex, contralateral motor cortex, and contralateral occipital lobe regions. The results showed that there was a significant positive correlation between the fApEn of EMG signal of the BIC and the HbO_2_ integral value of the contralateral motor cortex under the dominant side condition (r = 0.6268, *p* < 0.0001) (Figure 8B). In addition, the results showed that fApEn of EMG signal of the BIC was significantly positively correlated with HbO_2_ integral value in both contralateral prefrontal cortex (r = 0.6995, *p* < 0.0001) and contralateral occipital lobe (r = 0.4815, *p* = 0.0012) under the dominant side condition (Figures 8A, C). The same trend was observed during elbow isometric contractions of the non-dominant side. The results showed that the fApEn of EMG signal of BIC was also significantly positively correlated with the HbO_2_ integral value of the contralateral prefrontal cortex (r = 0.5949, *p* < 0.0001), contralateral motor cortex (r = 0.5091, *p* = 0.0006) and contralateral occipital lobe (r = 0.5749, *p* < 0.0001) under the non-dominant side condition (Figures 8D–F).

**FIGURE 9:**
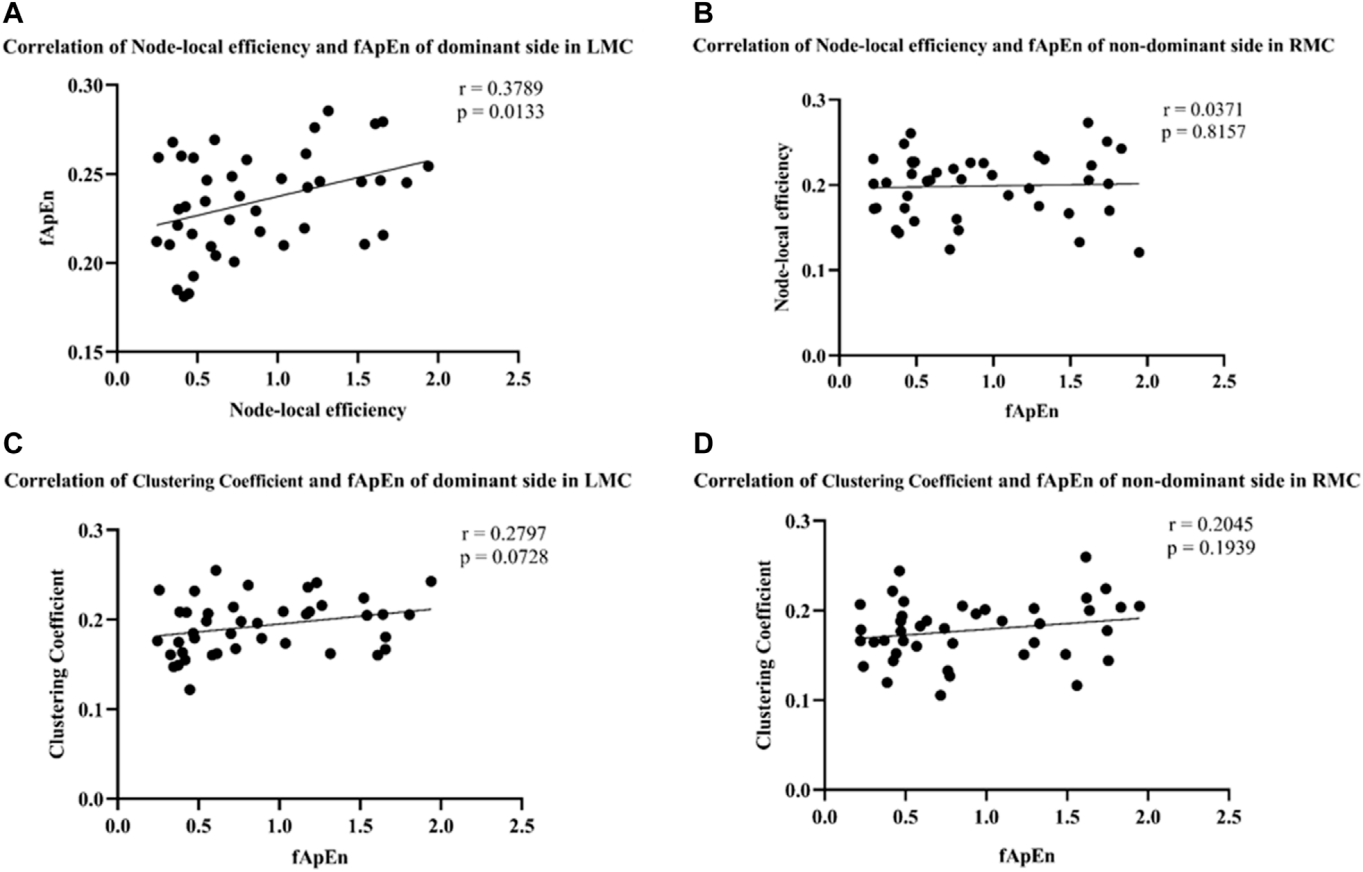
**(A,B)** Correlation of node-local efficiency and fApEn of the dominant side in LMC (r = 0.3789, *p* = 0.0133) and the non-dominant side in RMC (r = 0.0371, *p* = 0.8157). **(C,D)** Correlation of clustering coefficient and fApEn of the dominant side in LMC (r = 0.2797, *p* = 0.0728) and the non-dominant side in RMC (r = 0.2045, *p* = 0.1939).

fApEn of EMG signals and brain network related indicators, mainly including node-local efficiency and clustering coefficient were further analyzed. The results showed that fApEn of EMG signal of BIC was significantly positively correlated with node-local efficiency of contralateral motor cortex under the dominant side condition (r = 0.3789, *p* = 0.0133) (Figure 9A). However, no significant correlation was found between the fApEn of EMG signal of BIC and clustering coefficient of contralateral motor cortex under the dominant side condition (r = 0.2797, *p* = 0.0728) (Figure 9C). Similarly, no significant correlation was found between the fApEn of EMG signal of BIC and node-local efficiency (r = 0.0371, *p* = 0.8157) and clustering coefficient (r = 0.2045, *p* = 0.1939) of contralateral motor cortex under the non-dominant side condition (Figures 9B, D).

## 4 Discussion

In this study, the correlation between muscle coordination, cerebral cortex activation, and brain networks during isometric elbow contraction were investigated by simultaneously collecting and analyzing EMG and fNIRS signals. The results showed fApEn of EMG signals was significantly positively correlated with changes in HbO_2_ integral value in the contralateral brain region of the dominant and non-dominant sides. In addition, fApEn of EMG signals was significantly positively correlated with the changes in node-local efficiency of the contralateral motor cortex under the dominant side. The results also revealed that the effective connectivity between brain networks was significantly enhanced when the dominant side exerted muscle contraction, compared with that of the non-dominant side. This implies that there might be a mapping correlation between the central nervous system of the brain and peripheral muscle contractions during motor tasks.

### 4.1 The change in brain networks during motor tasks

Previous studies using fNIRS on relevant mechanisms of action in motor tasks have focused on brain region activation analysis (Lee et al., 2019; Shamsi and Najafizadeh, 2019; Shamsi and Najafizadeh, 2021; Kim et al., 2022). Based on the activation analysis of brain regions, the changes related to the brain network during exercise tasks and compared functional connectivity, effective connectivity, and graph theory analysis of the brain network under different motor tasks were investigated in current study. As shown in figure 2 and figure 3, it was found that functional connections between the prefrontal cortex, motor cortex, and occipital cortex were significantly enhanced in both dominant and non-dominant sides with greater contraction level, especially between the contralateral prefrontal cortex, ipsilateral prefrontal cortex, and contralateral motor cortex. Previous studies have shown the correlation between the complexity of neural activity in the brain and the complexity of the task caused by the level of force applied (Spraker et al., 2012; Alahmadi et al., 2015). The results of our study are consistent with those of previous studies.

Although many neuroimaging studies have analyzed handedness, the mechanism of the relationship between handedness and brain networks in motor tasks still needs to be fully understood (Klöppel et al., 2007; Lin et al., 2017). Many studies have shown that dominant hands perform better than non-dominant hands in motor tasks (Pool et al., 2014; Xiao et al., 2019). As shown in Figure 5, the results revealed that the contralateral motor cortex’s clustering coefficient and node-local efficiency increased significantly when the dominant side exerted force in a larger MVC. Meanwhile, under the same force conditions in this study, the effective connectivity of the brain network in the dominant side was significantly higher than that in the non-dominant side (Figure 4). In a review article from Hammond, the author also concluded the similar findings of the correlates of the handedness in primary motor cortex (Hammond, 2002). In addition, a fNIRS study from Yokoyama et al. showed that non-dominant side had greater variability in adjustment time than the dominant side under the same muscle relaxation and contraction conditions (Yokoyama et al., 2019). These findings suggest that there are significant differences in motor control between the dominant and non-dominant sides of healthy adults, and motor control might be more difficult on the non-dominant side.

### 4.2 The change in muscle control during motor tasks

Some of previous studies on approximate entropy or sample Entropy recommended using the standard deviation of segmented data for the determination of the r value (Zhou et al., 2011; Zhang and Zhou, 2012). But Sun and others asserted that the r value should be upheld as a universal constant for fuzzy approximate entropy analysis, with the aim of mitigating the impact of noise on the complexity of EMG signals (Sun et al., 2014). Figures 6C, D illustrate the response of tolerance r and fApEn and our results were similar with Sun’s finding and the N was set at 5,000 and r was fixed at 0.15. As shown in Figure 7, the study revealed that the fApEn of the EMG signals were significantly higher under the larger MVC force than under the smaller MVC force in both dominant and non-dominant sides, indicating more MUs were involved and more complicated motor firing pattern were triggered in the larger contraction force. Previous studies revealed the composition of EMG signals were the summation of a temporal superposition of the motor unit action potentials (MUAPs) sequence produced by the various motor units of the contracted muscles (De Luca, 1984; Gazzoni et al., 2004; Chen and Zhou, 2016). As the task intensity increased, the neuromuscular control strategy adjusted and structure in motor execution increased, leading to more complicated muscle firing patterns (Enders et al., 2015; Ma et al., 2017; Deng et al., 2021). As muscle power increases, more motor units are recruited, increasing the fApEn of the EMG signals (Kamavuako et al., 2012; Chen et al., 2018). Our results confirm that the complexity of BIC activation increases considerably with increasing strength levels, suggesting the motor control strategy may be adjusted accordingly.

The CCI can evaluate the degree of upper limb muscle activation and muscle coordination (Sheng et al., 2022). As shown in Figure 7, the results revealed that the CCI of EMG signals increased significantly in both dominant and non-dominant sides with higher MVC force. This may be because increased strength levels require more muscles to maintain strength output, leading to a significant increase in muscle co-contraction. A previous study reported that expert drummers reduced co-contraction in wrist flexor and extensor muscles (Beveridge et al., 2021). The lower CCI value hypothesized the for more exercised side qualified more precise and more efficient movement control.

### 4.3 The effects of corticomuscular interaction during motor tasks

As shown in Figure 8, we found that the fApEn of EMG signals was positively correlated with the changes in blood oxygen in contralateral motor cortex under both dominant and non-dominant sides (Figures 8B, E). Kumai et al. also found a significant correlation between brain activation and lower limb muscle activity during postural control tasks (Kumai et al., 2022). In addition, Liu et al. showed a substantial correlation between EEG and EMG and coherence in the beta band during both arm flexion and extension tasks (Liu et al., 2021). At high strength levels, muscles produce stronger contractions and activate more motor neurons (Xi et al., 2021). Therefore, the above findings might provide evidence that the higher the force intensity, the more numbers of motor units are involved in the motor task and correlated with the higher activation of corresponding brain regions. As the increase in the number of motor units leads to the increase in the density of the EMG signal, the non-linear characteristic (fApEn) of the EMG signal also increases. However, the number of motor units may not be the only factor for the variation of the non-linear characteristics of the EMG signal. The coherence of motor units and the firing rate may also change the variation of the EMG signal (Cashaback et al., 2013). McManus et al. showed a significant correlation between sEMG-based non-linear features and the underlying motor unit coherence. The results of our study are consistent with those previous studies (McManus et al., 2019). In addition, the motor control of the corresponding brain regions is strengthened, resulting in the increase of oxygen metabolism rate in local brain regions, thus enhancing the activation of the cerebral cortex (Leff et al., 2011). Takahashi et al. used a combination of EMG and fNIRS to study changes during muscle fatigue in healthy adults (Takahashi et al., 2021). The results showed that there was a correlation between peripheral muscles and cerebral blood oxygen during muscle fatigue, and the premotor cortex was specifically activated. The results of our study showed a consistent trend with this study (Takahashi et al., 2021). Another interesting finding from current study is that the fApEn of EMG was positively correlated with the changes of blood oxygen in the prefrontal and occipital cortex on both dominant and non-dominant sides (Figures 8A, C, D, F). Although many previous studies have suggested that motor tasks are primarily controlled by the motor cortex, the prefrontal cortex which is related to cognitive function may also be highly involved (Dayan and Cohen, 2011; Rizzolatti et al., 2014). Zou and coworkers’ finding also revealed that cognitive control plays an important role in complex motor tasks in a subject group of stroke patients (Zou et al., 2018). Since the task of 80% MVC is more challenging and the subject kept increasing their attention on screen to continuously follow the target line, it may cause the significant correlations between occipital cortex hemodynamics and complexity of muscle contraction. Therefore, all these findings provide evidence of a mapping relationship between the brain activity and the execution of motor tasks. The results also imply that exploring the coupling mechanism between the cerebral cortex and muscle contraction might provide a new evaluation method for rehabilitation intervention for motor function.

Previous studies have shown dynamic changes in corticomuscular coupling and brain network during muscle contraction (Siemionow et al., 2010; Xi et al., 2021). Similarly, we found that the fApEn of EMG signals was significantly positively correlated with changes in the node-local efficiency of the contralateral motor cortex (LMC) on the dominant side (Figure 9A). However, we did not find the significant relationship on the non-dominant side (Figure 9B). Furthermore, no significant correlation was found between the fApEn of EMG signals and clustering coefficient of the contralateral motor cortex on both dominant and non-dominant sides (Figures 9C, D). In previous studies of brain network, node-local efficiency reflects the integration ability and fault-tolerant ability of neighboring nodes (Li et al., 2013). This might mean that the brain network efficiency of contralateral motor cortex is still vital in the performance of motor tasks (Zimmermann et al., 2013). Clustering coefficient can be used to characterize the efficiency and ability of local information transmission in the network (Watts and Strogatz, 1998). However, due to the limited number of nodes in the brain network in this study, this might cause the non-significant correlation between node efficiency and muscle contraction tasks. By comparing Figures 8, 9, it indicated that the activations of local brain regions are different with the brain network node connections during elbow contractions. It means the connectivity between different brain regions might play a more important role to coordinate the completion of motor tasks than the local hemodynamic changes, but further evidence is needed (Thiebaut de Schotten and Forkel, 2022). Previous studies have also shown better recovery of local brain networks in stroke patients after completion of motor task training (Rehme and Grefkes, 2013; Xu et al., 2022). In general, our correlation results of brain network indicators demonstrated that the complexity of muscle contraction is significant related to the node-local efficiency of the contralateral motor cortex when performing isometric elbow contraction. This might provide a useful method for future clinical research to evaluate the rehabilitation of motor function and help us understand the mechanism behind these interventions.

### 4.4 Limitations

There were several limitations in the study. First, the experiment only recruited young, healthy people, and the interpretation may not be directly applied in older people and those with neurological conditions such as stroke. Second, the task of elbow isometric stretching was not set in the study, so the results did not address the task of the TRI as an active muscle to detect the co-contraction changes during elbow extension. In future studies, the relevant aspects of the elbow isometric stretch task will be further explored. Finally, only the cerebral hemodynamics in the time domain were analyzed, but not in the frequency domain. The physiological significance of cerebral hemodynamics in different frequency domains remains to be further studied.

## 5 Conclusion

In summary, this study demonstrated the changes of brain network related indexes and the non-linear characteristics of EMG signals under different motor task conditions, and explored the differences between dominant and non-dominant sides in motor tasks. The results found that effective connectivity between brain networks was significantly enhanced when the dominant side performed muscle contractions compared to the non-dominant side, which may imply that brain network analysis is a potential way to explore brain function during motor tasks. At the same time, fApEn of EMG signals increased significantly under greater MVC force, which was related to the increasing complexity of motor tasks. Finally, this study demonstrated a significant positive correlation between cerebral oxygen changes and EMG signals during motor tasks. This finding provides evidence for further exploration of the mapping relationship between the brain activity and the execution of motor tasks and can guide the movement function damage in patients with clinical rehabilitation.

## Data Availability

The datasets analyzed during the current study are available from the corresponding authors upon reasonable request.
